# Insulin-induced mTOR signaling and gluconeogenesis in renal proximal tubules: A mini-review of current evidence and therapeutic potential

**DOI:** 10.3389/fphar.2022.1015204

**Published:** 2022-10-10

**Authors:** Motonobu Nakamura, Nobuhiko Satoh, Shoko Horita, Masaomi Nangaku

**Affiliations:** Division of Nephrology and Endocrinology, The University of Tokyo, Tokyo, Japan

**Keywords:** mTOR, insulin resistance, proximal tubules, gluconeogenesis, diabetes

## Abstract

Energy is continuously expended in the body, and gluconeogenesis maintains glucose homeostasis during starvation. Gluconeogenesis occurs in the liver and kidneys. The proximal tubule is the primary location for renal gluconeogenesis, accounting for up to 25% and 60% of endogenous glucose production during fasting and after a meal, respectively. The mechanistic target of rapamycin (mTOR), which exists downstream of the insulin pathway, plays an important role in regulating proximal tubular gluconeogenesis. mTOR is an atypical serine/threonine kinase present in two complexes. mTORC1 phosphorylates substrates that enhance anabolic processes such as mRNA translation and lipid synthesis and catabolic processes such as autophagy. mTORC2 regulates cytoskeletal dynamics and controls ion transport and proliferation *via* phosphorylation of *SGK1*. Therefore, mTOR signaling defects have been implicated in various pathological conditions, including cancer, cardiovascular disease, and diabetes. However, concrete elucidations of the associated mechanisms are still unclear. This review provides an overview of mTOR and describes the relationship between mTOR and renal.

## Introduction

Gluconeogenesis occurs in the liver and renal proximal tubules (PTs), and the PTs produce a considerable amount of total glucose during fasting. Renal gluconeogenesis accounts for up to 60% and 25% of endogenous glucose production during fasting and after a meal, respectively (Alsahli and Gerich, 2017). Studies have demonstrated that knockout of insulin receptors in the PTs causes hyperglycemia, and insulin suppresses renal gluconeogenesis (Cersosimo et al., 1994; Cersosimo et al., 2000). Insulin and the mechanistic target of rapamycin (mTOR) regulate glucose transporter (GLUT4) translocation *via* a positive feedback loop between Akt2 and mTORC2. Moreover, several recent studies have reported that insulin regulates renal tubule functions (Nakamura, M. et al., 2020; Hoorn et al., 2020; Sohara et al., 2011; Chávez-Canales et al., 2013)

As such, insulin is an important regulator of PT gluconeogenesis (Humphrey et al., 2013), and the insulin/mTOR pathway has recently been shown to regulate PT gluconeogenesis (Nakamura et al., 2020a).

mTOR complex 1 (mTORC1) consists of mTOR, Raptor, GβL, and DEP domain-containing mTOR-interacting protein (DEPTOR) and is inhibited by rapamycin (Foster and Fingar, 2010). mTORC1 is a key growth regulator that senses and integrates a variety of nutritional and environmental factors, including growth factors, energy levels, cellular stress, and amino acids. In response to these signals, mTORC1 promotes cell proliferation by phosphorylating substrates that enhance anabolic processes, such as mRNA translation and lipid synthesis, or by limiting catabolic processes, such as autophagy. The second complex–mTORC2–is composed of mTOR, Rictor, GβL, SAPK interacting protein 1 (Sin1), PRR5/Protor-1, and DEPTOR. mTORC2 promotes cell survival by activating protein kinase B (Akt), regulating cytoskeletal dynamics *via* activation of PKCα, and regulating ion transport and proliferation *via* phosphorylation of *SGK1*. mTOR signaling defects are associated with many pathological conditions, including cancer, cardiovascular disease, and diabetes (Szwed et al., 2021). However, concrete elucidations of the associated mechanisms remain unclear. This review aimed to describe the current understanding of mTOR with respect to insulin and summarize the relationship between mTOR and the renal tubules in glucose metabolism.

## Regulatory mechanisms of mTOR complexes

**FIGURE 1:**
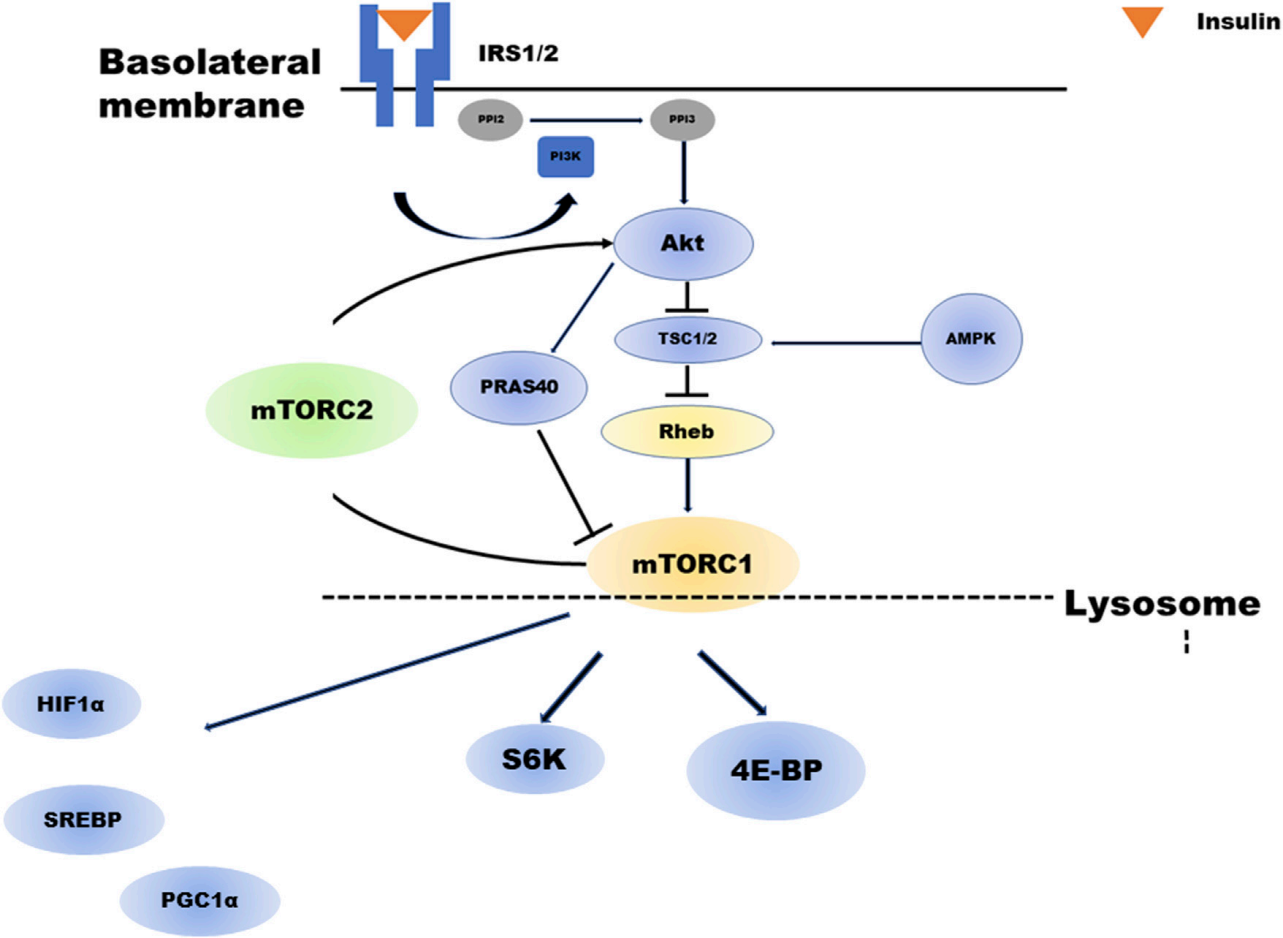
Overview of the mTOR cascade. Insulin activates both mTORC1 and mTORC2. Akt regulates mTORC1. TSC2 phosphorylated by insulin-activated Akt will have its activity suppressed. As a result, the activity of GTP-bound Rheb increases, and mTORC1 is activated. In addition, Akt phosphorylates PRAS40, which in turn activates mTORC1. mTORC1 regulates several negative feedback loops that control IRS1/2 and mTORC2 signaling: direct inhibition of IRS1/2 and indirect inhibition of mTORC2 *via* S6K1. abrAbbreviationsmTORC; mechanistic target of rapamycin complex, Akt; Protein kinase B, TSC; tuberous sclerosis complex, PRAS40; proline-rich Akt substrate of 40 kDa, IRS; insulin receptor substrate, PIP2; Phosphatidylinositol 4,5-bisphosphate, PIP3; Phosphatidylinositol 3,4,5-trisphosphate.

Figure 1 depicts a brief overview of the mTOR cascade. Ras homolog enriched in brain (Rheb), a small GTP-binding protein that has been shown to mediate cell growth and control cell size in mammalian cells, is a potent stimulator required for mTORC1 kinase activity in its GTP-bound state and is negatively regulated by the heterodimer tuberous sclerosis complex (TSC1/2) of the GTPase-activating proteins (GAP-TSC) (Fukuda and Shiozaki, 2018; Zhu and Wang, 2020). Much of the upstream stimulation is transmitted through Akt and TSC1/2, which regulate the nucleotide loading state of Rheb (Tee et al., 2003). In contrast, amino acids signal independently from the PI3K/Akt pathway to mTORC1. Particularly, amino acids facilitate the translocation of mTORC1 to the lysosomal surface, where mTORC1 is activated by binding to Rheb. This process is regulated by the concerted action of multiple complexes, mainly v-ATPase, Ragulator, Rag GTPase, and GATOR1/2 (Saxton and Sabatini, 2017; Wolfson and Sabatini, 2017). The enhancement of anabolism by mTORC1 occurs when insulin is present, this cascade is strongly dependent on feeding. Feeding actives mTORC1, whereas during fasting inhibits to conserve limited resources (Saxton and Sabatini, 2017). The process of mTORC1 activation is outlined ahead.

mTORC1 activation is initiated by the low-molecular weight GTP-binding protein Rheb. Rheb activity is regulated by GAPs or TSC1-TSC2 complexes, inhibitory regulators for Rheb. TSC1 and TSC2 are essential for the stability of the TSC2 protein, which has a GAP domain and forms a complex with TSC1. Phosphorylation of TSC2 by insulin-activated Akt suppresses its activity. Consequently, Rheb activity increases (GTP-bound Rheb increases), and mTORC1 is activated. In addition, Akt phosphorylates PRAS40, a component of mTORC1, in turn activating mTORC1.

TSC2 is also phosphorylated by extracellular signal-regulated kinase 1/2 and GSK3β, and its activity is suppressed, resulting in mTORC1 activation. In contrast, depletion of intracellular ATP activates AMP-dependent kinase (AMPK). It is reported that even if Rheb activity increases in the presence of cell growth factors such as insulin and IGF-1, mTORC1 cannot be activated without the amino acid. Rheb is expressed on late endosomal membranes in the cell but cannot recruit mTORC1 from the cytoplasm; thus, mTORC1 cannot be activated without the amino acid. Recently, it has been demonstrated that another GTP-binding protein, Rag, plays a role in this recruitment in an amino acid-dependent manner (Sancak et al., 2010; Zhu and Wang, 2020).

Several studies have shown that mTORC2 is regulated by factors such as phosphatidylinositol-3,4,5-triphosphate (PIP3)-mediated growth factor (Liu et al., 2015), Sin1, phosphorylation (Liu et al., 2013), GβL-ubiquitination (Wang et al., 2017), and reactive oxygen species (Bernard et al., 2020). However, regarding gluconeogenesis in PTs, the molecular mechanism of mTORC2 activation remains largely unknown.

## Regulatory mechanisms of mTORC2

Nutrients and growth factors also influence mTORC2 activation, but how they regulate mTORC2 is more complex and unknown compared to mTORC1. In mammals, increased growth factor/PI3K signaling enhances mTORC2 activity. PIP3 activates mTORC2 at the membrane, and mTORC2 may be regulated in different membrane compartments. The endosomal pool of mTORC2 mediates the PI3K signaling pathway (Ebner et al., 2017), and insulin/PI3K increases mTORC2 activation and binding to ribosomes (Zinzalla et al., 2011). The localization of compartments is pivotal in regulating this endosomal pool (Jia and Bonifacino, 2019). In addition, mTORC2 responds to intracellular nutrient or metabolite status. Activation of mTORC2 is enhanced by removing amino acids (e.g., glutamine) and glucose starvation in the presence of serum (Moloughney et al., 2016). Activation of mTORC2 during glutamine or glucose starvation is essential to maintain flux through the hexosamine biosynthetic pathway. During glucose depletion, AMPK directly activates mTORC2 *via* phosphorylation of mTOR and rictor in the presence of PI3K signaling (Kazyken et al., 2019).

## mTORC1 and mTORC2 constitute a negative feedback loop

As shown in Figure 1, mTORC1 regulates several negative feedback loops that control IRS1/2 and mTORC2 signaling (i.e., directly inhibiting IRS1/2 and indirectly inhibiting mTORC2 *via* S6K1). In β-cell, the mTORC1-S6K1-mediated negative loop constitutes an intracellular regulatory mechanism that suppresses excessive insulin and mTORC2 signaling under physiological conditions and controls cell growth and homeostasis. Meanwhile, chronic mTORC1-S6K1 hyperactivation induced by obesity and overnutrition may attenuate insulin and mTORC2 signaling, resulting in decreased β-cell function and type 2 diabetes (T2D) development. These pathways are not limited to pancreatic beta cells and may also be involved in proximal tubular metabolism (Ardestani et al., 2018).

## Roles of rheb in the regulation of gluconeogenesis through the insulin/mTOR pathway

The key factors in the insulin/mTORC1 pathway are the TSC1/TSC2 complex and Rheb. Recently, the mechanism by which mTORC1 is activated by Rheb and inhibited by proline-rich Akt substrate of 40 kDa (PRAS40) was demonstrated. Rheb is an activator of mTORC1, and GTP-bound Rheb (activated form) binds directly to the kinase catalytic domain of mTORC1 to enhance mTORC1 kinase activity (Long et al., 2005). Further, Rheb increased the binding of mTORC1 to its substrate, 4EBP1 (Sato et al., 2009), and insulin-activated Akt directly phosphorylated TSC2. This phosphorylation directly decreases the GAP activity of TSC2 or changes its subcellular localization, resulting in decreased TSC1/TSC2 activity and consequently increased GTP-bound Rheb (Li et al., 2004). Furthermore, activated Akt activates the mTORC1 pathway by inhibiting PRAS40, an inhibitor that directly binds mTORC1 (Haar et al., 2007; Sancak et al., 2007). PRAS40 has a TOR signaling (TOS) motif found in substrates of mTORC1, and its inhibition is thought to be due to competition for mTORC1 substrates. Only a few proteins are known to be direct substrates of mTORC1, such as 4EBP1, S6K1, STAT3, and Hypoxia Inducible Factor (HIF)1α (Szwed et al., 2021).

## Regulation of GLUT4 by the insulin/mTOR pathway

In the absence of insulin stimulation, GLUT4 is deposited in intracellular vesicles, but stimulation occurs when insulin binds to its receptor on the plasma membrane, and GLUT4 translocates to the plasma membrane, taking up glucose from the blood into the cell. Intracellular signaling triggered in response to insulin is complex, and Akt, activated by insulin, is phosphorylated by several substrates. This is essential for regulating insulin-dependent processes (Lizcano and Alessi, 2002). In addition, Akt phosphorylates and inhibits AS160, a Rab-GTPase-activating protein. It promotes the translocation of GLUT4 to the plasma membrane by activating the Rab small GTPase and regulating cytoskeletal reorganization (Sano et al., 2003). Furthermore, Akt phosphorylates forkhead box protein O (FOXO), a transcription factor necessary for the expression of gluconeogenic and lipogenic enzymes. For example, FOXO1 activates gluconeogenic genes in the liver (Puigserver et al., 2003), negatively regulates *SREBP1c* (Kamei et al., 2008), and inhibits lipogenesis (Nakae et al., 2003). Akt activated by insulin phosphorylates serine 256 of FOXO1, inhibiting the expression of gluconeogenic enzymes. Moreover, Akt modulates mTORC1 signaling by phosphorylating and inhibiting TSC1/2, a negative regulator of mTORC1 (Shimobayashi and Hall, 2014). Subsequently, mTORC2 regulates glucose homeostasis *via* Akt. Akt promotes glucose uptake by increasing GLUT4 translocation to adipocyte membranes (Kohn et al., 1996). Additionally, Akt phosphorylates and deactivates GSK-3 and reduces the phosphorylation rate of glycogen synthase. As a result, glycogen synthase activity increases, leading to increased glycogen accumulation in the muscle and liver (Manning and Cantley, 2007). Akt also regulates glucose homeostasis by phosphorylating and inhibiting FOXO1, a transcription factor that regulates gluconeogenesis (Accili and Arden, 2004).

## Therapeutic potential of insulin/mTOR regulation of gluconeogenesis

mTORC1 plays an important role in the development and progression of β-cell damage in T2D. It is possible that mTORC1 functions as a “double-edged sword” by its regulation of β-cell volume and function in response to metabolic stresses (Ardestani et al., 2018). In fact, hyperlipidemia occurs in 75% of patients treated with mTOR inhibitors (Busaidy et al., 2012; Pallet and Legendre, 2013). Surprisingly, 32% of participants treated with everolimus develop hyperglycemia and IR, resulting in new onset of diabetes (Kaplan et al., 2014). Meanwhile, increased mTOR activity is associated with IR (Mordier and Iynedjian, 2007; Hsu et al., 2011), and short-term treatment with caloric restriction and rapamycin leads to increased insulin sensitivity and glucose uptake, suggests the contradictory roles of mTOR and mTOR inhibitors (Ye et al., 2012).

Thus, the chronic effects of rapamycin on insulin sensitivity and glucose metabolism *in vivo* remain elusive. Chronic rapamycin treatment promoted IR, severe glucose intolerance, and increased gluconeogenesis in a rat model. It was also linked to increased expression of the gluconeogenic enzymes or genes phosphoenolpyruvate carboxykinase (*PEPCK*) and G6Pase in the liver, increased expression of the transcriptional coactivator peroxisome proliferator-activated receptor-γ coactivator-1α (PGC-1α), and enhanced nuclear mobilization of FOXO1, CRTC2, and CREB. These changes were observed despite normal activation of the IRS/PI3k/Akt pathway in the liver of rapamycin-treated rats (Houde et al., 2010). These results demonstrate that chronic inhibition of the mTORC1/S6K1 pathway by rapamycin causes impaired glucose tolerance by inducing transcriptional activation of gluconeogenic genes, primarily through coordinated activation of PGC-1α, CRTC2, CREB, and FOXO1.

Recently, it was suggested that Rheb, which plays a vital role in the insulin/mTOR pathway, is associated with a mechanism by which IR improves in patients undergoing duodenal-jejunal bypass (DJB). Individuals who received DJB demonstrated improved hepatic IR and better insulin signaling, resulting in better control of glucose tolerance. Furthermore, transfection of Wistar rats treated with DJB with a miR-200a inhibitor increased Rheb protein levels and enhanced the feedback effect on insulin receptor substrate-dependent insulin signaling. Meanwhile, transfection with a miR-200a mimic caused the opposite effect. Furthermore, downregulation of miR-200a decreased insulin sensitivity, whereas upregulation of miR-200a in diabetic rats improved diabetes. These results indicate that improvement of hepatic IR is closely associated with Rheb-targeted miRNA-200a. These results could explain the crosstalk between miRNAs and insulin signaling pathways in bariatric surgery (Guo et al., 2016).

## Renal tubular effects of insulin

The kidneys remove approximately 35% of the secreted insulin. Most insulin is filtered through the glomerulus and completely reabsorbed from the PT’s brush border membrane (BBM). Upon release, insulin is trafficked through endocytic vesicles to the vacuole, where it is disassembled (Kolman et al., 2009). Endocytosis occurs after insulin binds to the Megalin-cubilin complex, which binds to the insulin receptor (IR) present in the BBM of the PT. Megalin is a transmembrane complex protein that salvages most of the serum proteins, including insulin, and is abundantly expressed in the PT segment S1 (Orlando et al., 1998; Bryniarski et al., 2018). Insulin increases its uptake and degradation by inducing an increase in megalin content (Hosojima et al., 2009). Less than 1% of the filtered insulin is transcytosed to the basolateral membrane (Nielsen, 1993), and only 1% is excreted in the urine eventually. Insulin reaches its highest concentration in the PT, where it effects gluconeogenesis suppression (Gatica et al., 2013) and sodium reabsorption (Nakamura et al., 2015a; Nakamura et al., 2020b). Additionally, insulin acts on other tubular sites where IR is abundantly expressed.

## Regulatory mechanisms of proximal tubular glucose transporters

PT cells can generate large amounts of energy using β-oxidation of fatty acids and are capable of producing glucose through gluconeogenesis. PTs do not exhibit glycolytic capacity except in the S3 segment; these cells depend on oxidative phosphorylation for metabolism and are able to produce glucose by gluconeogenesis. In acute kidney injury, PT metabolism shifts from fatty acid β-oxidation and gluconeogenesis to glycolysis, whereas in chronic kidney disease, fatty acid oxidation is lost (Faivre et al., 2021; Scholz et al., 2021). Glucose transport in PT is mediated by GLUT and SGLT. GLUT is abundantly expressed in the kidney and is a cell surface glucose transporter. GLUT1 expression is located in the PT S3, TAL, and collecting ducts, and GLUT2 expression is located in PT S1. In cultured mouse PT, insulin increased GLUT1 mRNA, membrane protein content, and glucose uptake (Kang et al., 2010). In HEK cells, GLUT1 traffic to the apical membrane has also been demonstrated under PI3K/AKT signaling with increased glucose uptake (Zambrano et al., 2019). Note that mTOR has recently been implicated in regulating GLUT1 in cancer cells and has been considered a potential therapeutic target, although the association between mTOR and glucose transporters in the PT remains unclear.

Regarding GLUT2, plasma and luminal glucose concentrations have been shown to increase the expression of GLUT2 and translocate it from the basolateral to the BBM side in animal models (Marks et al., 2003). GLUT2 studies in humans have shown that GLUT1 and GLUT2 expressions are remarkably decreased in patients with T2DM compared to those in controls, although there are many unknowns regarding its localization (Norton et al., 2017; Solini et al., 2017).

For GLUT4 trafficking, insulin-dependent GLUT4 trafficking through Ins/Akt signaling occurs in the PTs (Mendes et al., 2010; Coffey et al., 2015), as well as in adipocytes (Sano et al., 2003) and myocytes (Bruss et al., 2005). Although it is clear that the Insulin/mTOR pathway regulates GLUT4 in several cell experiments, whether mTOR pathway also regulates GLUT4 in the PTs remains unclear.

SGLT transports glucose against its concentration gradient using an electrochemical sodium gradient. SGLT1 is found along all PT segments, abundantly expressed in S3 and the outer medulla than in the cortex (Balen et al., 2008). In contrast, SGLT2 is abundantly expressed in the renal cortex, especially in the S1 and S2 segments (Tojo et al., 2015). Insulin regulates SGLT1 directly (Ghezzi and Wright, 2012) and indirectly (Saad et al., 2005). However, there are conflicting reports on SGLT2 regulation. In cultured human renal cells, SGLT2 activity (Ghezzi and Wright, 2012) and protein levels (Nakamura et al., 2015b) were increased independent of insulin and glucose. In contrast, Na^+^ glucose transport increased by 250% by insulin in HEK cells, while hSGLT1 was relatively insensitive to insulin (Ghezzi and Wright, 2012). Thus, there is still no established theory on the insulin-mediated regulatory mechanism of SGLT, and the relationship between the Insulin/mTOR pathway and SGLT in the PTs remains unclear.

## Gluconeogenesis in kidneys and the role of mTOR

The kidneys, unlike the liver, cannot store large amounts of glycogen and produce glucose through gluconeogenesis. Renal gluconeogenesis is negatively regulated by insulin (Tiwari et al., 2013), while catecholamines, cortisol, thyroid hormone, growth hormone, and parathyroid hormone are positive regulators (Stumvoll et al., 1997; Meyer et al., 2003).

The role of mTORC1 in insulin signaling is multifactorial (Laplante and Sabatini, 2012); mTORC1 induces PPARγ in adipose progenitor cells, promoting their differentiation into adipocytes, in turn leading to fat accumulation (Shan et al., 2016; Soliman 2011). S6 kinase activated by mTORC1 forms a feedback loop by phosphorylating insulin receptor substrate (IRS)1, which mediates insulin receptor signaling, thereby suppressing IRS1 function. This mechanism reportedly plays a vital role in IR. Akt activated by mTORC2 phosphorylates the transcription factors FOXO1 and PPAR to suppress their function. FOXO1 induces PEPCK in the liver. Therefore, mTORC2 may inhibit gluconeogenesis in the liver (Dentin et al., 2007).

Gluconeogenesis occurs in the PTs and liver. mTORC1/2 in PTs may have a different role than the liver. In the liver, insulin regulates gluconeogenesis through the IRS2/PI3K/Akt/FOXO1 pathway (Cheng et al., 2010). Insulin has been reported to suppress gluconeogenesis in PT and the liver (Tiwari et al., 2013). Gluconeogenesis, regulated by insulin, plays an essential role in IR and achieving therapeutic objectives in T2D (Hatting et al., 2018). Indeed, renal gluconeogenesis is enhanced in animal models. Elsewhere, high postprandial insulin levels have been shown to decrease transcription of PT gluconeogenic enzymes in mice (Sasaki et al., 2017) and rabbits (Usarek et al., 2014), and the expression of gluconeogenic genes is reduced by glucose counterregulatory effects in cases of IR and deficiency. Furthermore, primary cultured cells obtained from the kidneys of participants (Pandey et al., 2017) and HK2 cell (Sasaki et al., 2017) exposed to insulin undergo gluconeogenesis reduction.

We recently reported that insulin directly inhibits gluconeogenesis in PTs and that the inhibitory effect of insulin on gluconeogenesis is mediated by the IRS1/Akt2/mTORC1/2 pathway (Nakamura et al., 2020a). Additionally, increased gluconeogenesis in PTs due to mTORC1 and mTORC2 inhibition could contribute to hyperglycemia. It is generally accepted that mTORC1 negatively regulates insulin signaling *via* S6K phosphorylation in nutrient-saturated states (Um et al., 2004). Prolonged exposure to rapamycin, an mTORC1 antagonist, induces insulin intolerance and hyperglycemia, which has been attributed to mTORC2 inhibition rather than mTORC1 in the liver (Lamming et al., 2012). Moreover, a recent study using a mouse model reported that mTOR was differentially involved in tubular autophagy activity *via* the insulin/mTOR pathway in the presence of nephropathy, depending on the type of diabetes. This study showed that insulin suppressed autophagy, whereas glucose loading increased it. In T1D, autophagic activity was enhanced and more protective than expected because of hyperglycemia and low insulin; in contrast, in T2D, hyperinsulinemia suppressed autophagy induction (Sakai et al., 2019).

## mTOR and inflammation

Regarding inflammation, our current understanding of the regulatory mechanisms of mTORC2 is limited. However, mTORC1 and mTORC2 promote T-cell reprogramming, B-cell proliferation, and antibody responses. In macrophages, inhibition of mTOR activity or glycolysis decreases the production of proinflammatory cytokines and the ability to eliminate bacteria. Further, in response to microbial stimuli (e.g., β-glucan), classically-activated macrophages increase aerobic glycolysis *via* mTORC1, which regulates HIF1. Therefore, it plays an important role in immune cell regulation but is independent of insulin signaling. mTORC1 is potently activated by amino acids, growth factors, T-cell receptors, and interleukin 2.

Regulatory T-cells are strongly regulated by mTORC1, and mTORC1 suppression is known to have immunosuppressive effects (Zeng and Chi, 2017). Based on these mechanisms, therapeutic applications of mTOR inhibitors on inflammation have been investigated. In fact, sirolimus completely suppressed autoimmunity in lupus-prone mice (Warner et al., 1994) and suppressed disease activity in a retrospective study of systemic lupus erythematosus (SLE) participants (Caza et al., 2014). Furthermore, in a recent clinical trial in active symptomatic SLE participants, 12 months of sirolimus treatment was associated with improvement in disease activity, which was suggested to be associated with modification of inflammatory T-cell lineage specification (Lai et al., 2018). In addition to these findings, it has recently been shown that mTORC1 activation in glomeruli is higher in participants with lupus nephritis than in participants with DMN (Mao et al., 2022), that *ATG5* and *ATG7* and the HRES-1/Rab4A are associated with the molecular pathogenesis of lupus, and genetically driven mTOR activation is associated with fulminant lupus nephritis (Caza et al., 2022).

## Discussion

In this review, we describe the recent findings regarding the relationship between mTOR and renal tubular gluconeogenesis in glucose metabolism. The roles of mTOR in insulin signaling are diverse. The insulin/mTORC1 feedback loop mechanism has been reported to play an essential role in IR. Insulin signals through PI3K; it regulates signaling to mTORC1 through Akt phosphorylation. In addition, Akt, activated by mTORC2, phosphorylates FOXO1 and PPARs, suppressing their function. Therefore, this regulatory mechanism is thought to suppress gluconeogenesis. This mechanism is also active in PTs wherein mTORC1 and mTORC2 are essential for regulating glyconeogenesis and closely involved in regulating acid–base equilibrium and Na transport *via* v-ATPase, NHE3, and NBCe1. Furthermore, a recent study reported that mTOR was differentially involved in tubular autophagy activity *via* the insulin/mTOR pathway in the presence of nephropathy, depending on the type of diabetes. (Sakai et al., 2019).

The regulatory mechanisms of mTORC2 remain unclear. In particular, the mechanism of mTORC2 activation during glutamine withdrawal remains to be elucidated but may involve sestrin2. Sestrin2 upregulates the catalytic activity of mTORC2, causing Akt Ser473 phosphorylation. Sestrin2 promotes Akt phosphorylation through Sestrin/GATOR2 and GATOR2/mTORC2 interactions (Kowalsky et al., 2020; Szwed et al., 2021). Thus, activation of mTORC2 during glutamine starvation may be associated with sestrin2-mediated repression of mTORC1. Sestrin3 is also associated with rictor and enhances mTORC2 activation in response to insulin and nutrient stimulation (Tao et al., 2015; Szwed et al., 2021).

The relevance of Rheb, a critical factor in the insulin/mTORC1 pathway in the PTs, remains unclear. mTORC1 enhances the expression of the rate-limiting enzyme of gluconeogenesis *via* PGC-1α; whether this pathway is also useful in humans and its physiological role in the PTs is also unknown. Inhibition by PRAS40 is thought to be because of competition for substrates of mTORC1, and proteins known to be direct substrates of mTORC1 include 4EBP1, S6K, STAT3, and HIF1α (Szwed et al., 2021). Given that Rheb is involved in substrate association, thus, insulin-induced mTORC1 activation may phosphorylate different substrates compared to amino acid stimulation, which may be associated with PT gluconeogenesis.

Long-term administration of rapamycin to rats promoted IR, severe glucose intolerance, and increased gluconeogenesis (Houde et al., 2010). These changes were observed in the liver of rapamycin-treated rats despite normal activation of the IRS/PI3K/Akt pathway. These results indicated that mTORC1/S6K could regulate gluconeogenesis by modulating several important transcription factors. The mechanism by which DJB improves IR may be related to Rheb, which plays a vital role in the insulin/mTOR pathway. Briefly, DJB may improve insulin resistance through Rheb suppression upstream of mTORC1/S6K (Guo et al., 2016). Although its involvement in PT gluconeogenesis is currently unknown, Rheb inhibition represents a potential new therapeutic target for diabetes and IR.

The effect of insulin on gluconeogenesis suppression differs in the liver and kidney. In the liver, SREBP1c directly suppresses IRS2 transcription and inhibits hepatic insulin signaling (Ide et al., 2004; Tsunekawa et al., 2011). Unlike the liver, the renal cortex is incapable of insulin-induced changes in the expression of IRS2 and SREBP1c (Nakamura N. et al., 2015). Even IR did not change SREBP1c expression in the renal cortex. This indicates that the regulation of IRS2 expression in the renal cortex is quite different from that in the liver. Therefore, the mechanism of gluconeogenesis regulation by the Insulin/mTOR pathway may also differ in the liver and PT.

A recent study showed that mTORC1 might be activated by sensing S-adenosyl methionine (SAM). According to this report, increased catabolism of SAM *via* the action of glycine N-methyltransferase could extend the lifespan of Drosophila (Obata and Miura, 2015). However, in a cross-sectional study of individuals with metabolic syndrome, plasma SAM levels were reported to be associated with increased fasting insulin levels, IR, and tumor necrosis factor-α (Lind et al., 2018). These results indicate conflicting conclusions, namely insulin mediates mTORC1 through SAM and that SAM concentrations are associated with IR. Although there is no unified perspective, a recent review suggested that selective suppression of mTORC1 may be a potential therapeutic target for aging, metabolic disorders, or aging-related diseases (Kitada et al., 2020). Despite many inconsistencies and unresolved aspects regarding insulin’s regulatory mechanisms on the mTOR signaling pathway, it is clear that mTOR regulation plays a very important role in regulating tubular gluconeogenesis and sodium transport, as earlier described in this review.

**FIGURE 2:**
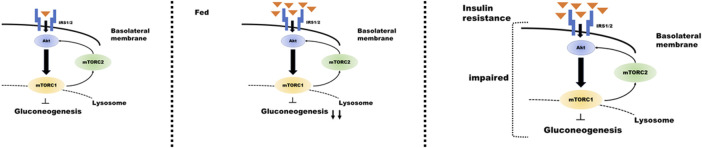
Regulation of insulin signaling in the proximal tubule during gluconeogenesis under different conditions. Proximal tubular gluconeogenesis responds differently depending on the state of insulin secretion and the presence of insulin resistance. When insulin is secreted after consuming food (center panel of the figure), insulin/mTOR signaling is enhanced, and gluconeogenesis is inhibited. The condition of insulin resistance (right panel) results in impaired insulin/mTOR signaling and enhanced gluconeogenesis despite high blood glucose levels.

Renal gluconeogenesis plays an essential role in normal physiological function, and its impairment has adverse pathological implications. Overall, this review elucidates that mTOR plays an important role in regulating renal gluconeogenesis. Signaling through the insulin/Akt pathway plays an important role in the regulatory mechanism of mTORC1 and mTORC2 in tubular gluconeogenesis; feeding enhances insulin secretion and suppresses proximal tubular gluconeogenesis. By contrast, in the presence of pathologies, such as IR or T2D, this signaling could be attenuated by downregulation of IRS2 expression (Nakamura et al., 2015a; Nakamura et al., 2020b). Furthermore, it suggests that renal gluconeogenesis is enhanced during IR. Such impairment may further contribute to hyperglycemia in T2D (Figure 2). However, further research is required in this area to explain the relevant mechanisms.

## Abbreviations

GAP, GTPase-activating proteins; Sin 1, SAPK interacting protein one; SAM, S-adenosyl Methionine; PT, Proximal tubules; mTOR, Mechanistic target of rapamycin; AMPK, AMP-activated protein kinase; Rheb, Ras homolog enriched in brain; IR, nsulin resistance.
